# A Case of Methanol Poisoning in a Child

**DOI:** 10.1155/2014/652129

**Published:** 2014-01-06

**Authors:** Reyner Loza, Dimas Rodriguez

**Affiliations:** ^1^Unit of Pediatric Nephrology, Department of Pediatrics, Cayetano Heredia National Hospital, Avenida Honorio Delgado 265, SMP, Lima 31, Peru; ^2^Cayetano Heredia University, Avenida Honorio Delgado 430, SMP, Lima 31, Peru; ^3^Unit of Pediatric Nephrology, Sergio Bernales National Hospital, Avenida Tupac Amaru 8000, Comas, Lima 7, Peru

## Abstract

We report the case of a girl admitted to the emergency room with a history of four hours' acute illness, characterized by nausea, vomiting, salivation, headache, blurred vision, and acidotic “Kussmaul” breathing. Arterial blood gases showed severe mixed acidosis, metabolic and respiratory with high anion gap. She had ingested the contents of a scent bottle containing methanol, which she thought was a soft drink bottle. The girl was managed with hemodialysis and strong intravenous hydration. She improved well and made a full recovery.

## 1. Introduction

Methanol poisoning in children is rarely described in the literature; some cases are reported as accidental ingestion. For this reason, we report here a case of accidental methanol poisoning, discussing clinical and laboratory manifestations, management, and evolution.

### 1.1. Case Report

A six-year-old female patient was admitted to the emergency room with her mother after four hours of disease characterized by nausea and vomiting of food content, abdominal pain, difficulty in breathing, salivation, headache, blurred vision, and psychomotor agitation. A physical examination found the following: weight 22 kg, blood pressure 80/60 mmHg, respiratory rate 32 breaths per minute, and heart rate 148 beats per minute.

Her skin was pale, and her eyes were sunken, underactive, clouded, and irritable to stimulus.

The patient was initially treated for severe dehydration resulting from food poisoning. However, with the development of wheezing and unresponsiveness to stimuli, she was transferred to the shock trauma unit for worsening respiratory distress, deep breathing with panting (Kussmaul) breathing, unresponsiveness to stimuli, Glasgow 10, to receive ventilator support.

The laboratory findings were as follows: yellow urine, specific gravity 1.025, pH 7.0, trace glucose, leukocytes 8−10x field, erythrocytes 2-3x field, the leukocyte blood count 8,180x mm^3^, segmented 69%, eosinophils 5%, lymphocytes 26%, Hb 12 g/dL, sodium 133 mEq/L, potassium 6 mEq/L, chloride 107 mEq/L, aspartate aminotransferase 4490 IU/L, alanine aminotransferase 8030 IU/L, and lactate dehydrogenase 2609 UI/L.

Arterial blood gases showed severe mixed acidosis, metabolic and respiratory with high anion gap (pH 6.9, PaO_2_: 108 mmHg, PaCO_2_: 26 mmHg, and HCO_3_: 3 mEq/L). We therefore assumed the possibility of diabetic ketoacidosis, salicylate poisoning, or methanol poisoning. Evaluation of renal function showed urea 33 mg/dL and creatinine 0.6 mg/dL; glucose was normal. Therapy was initiated with vigorous hydration with sodium chloride 9/1000 and supplemental intravenous sodium bicarbonate. The toxicology results showed a serum methanol of 1.47 mg/dL. Emergency hemodialysis therapy was initiated; the patient was dialyzed for an hour for two sessions.

The family gave us new information that the girl regularly took a drink called Kola Ingles. They stated that the patient had found a 250 mL pink perfume bottle and that she had ingested 200 mL of its contents, thinking it was the cola drink.

The patient improved progressively after hemodialysis with correction of her metabolic acidosis, liver function tests, and lactic dehydrogenase (Tables 1 and 2).

The child was discharged from the hospital in five days recovering full health.

## 2. Discussion

Methanol (CH_3_OH), also known as methyl alcohol, wood burning alcohol, or carbinol, is highly toxic. It is the simplest of the alcohols used in paints, varnishes, solvents, perfumes, plastic manufacture, photographic materials, antifreeze, and household cleaning products.

The pathways for poisoning are inhalation, cutaneous, and digestive tract, in most cases by swallowing. Methanol poisoning in children is rare and there are only isolated reports of homicidal poisoning and seizures [1, 2].

In some European countries, it is reported that the main cause of hospitalization among teens is alcohol poisoning. Reasons range from recreational use to poisoning and self-harm [3].

Sometimes symptoms may mask an underlying condition, which may delay diagnosis [4].

Individual or collective poisoning is usually voluntary or accidental ingestion in the case of adulterated liquor.

The stages of intoxication are described as follows. In the first phase, there is minimal decrease in central nervous system activity, weakness, dizziness, and nausea. The second phase is marked by the development of metabolic acidosis characterized by vomiting, abdominal pain, confusion, visual disturbances, photophobia, blurred vision, bilateral mydriasis, unresponsiveness to light, and occasional blindness. In the third phase, in direct relation to the degree of metabolic acidosis, neuronal injury occurs with retinal necrosis and hemorrhage in the basal ganglia of the brain. At this stage there is hypotension, coma, and Kussmaul breathing. Our patient was considered to be in the second phase of methanol toxicity.

Diagnosis of methanol poisoning is based on the suspicion of ingestion, the presence of visual disturbances, the onset of metabolic acidosis with elevated anion and osmolar gaps, and markedly increased liver enzymes.

Confirmation is by determining the plasma levels of methanol. The toxic methanol dose is 10–30 mL (100 mg/kg), although lower intakes have caused blindness. It is lethal above 60–240 mL (340 mg/kg). A dose of 30 mL of 100% methanol can be considered fatal.

Concentrations above 0.2 g/L are toxic, values higher than 0.5 g/L indicate severe poisoning, and concentrations above 0.9 g/L are potentially deadly.

Methanol is rapidly absorbed from the gastrointestinal tract, giving peak plasma levels after 30–90 minutes. The serum half-life ranges from 14 to 30 hours and is distributed freely. The kidney, in untreated patients, removes less than 5%.

In the liver, methanol is removed by biotransformation via alcohol dehydrogenase (ADH), forming formaldehyde, and subsequently through conversion of the aldehyde dehydrogenase to formic acid.

That methanol by itself is not toxic. It is the degradation of methanol by alcohol dehydrogenase that releases the toxic metabolites, formaldehyde, and formic acid.

The rational for treatment is reducing the formation of the toxic metabolites, by cleaning in hemodialysis or administering ethanol, Fomepizole.

Hemodialysis treatment removes the methanol and his metabolites from the circulation, reducing the shelf life. This is preferable to peritoneal dialysis as it results in more rapid clearance. Further, ethanol is a treatment form that competitively inhibits the metabolism of methanol by alcohol dehydrogenase. The affinity of the enzyme for ethanol is 10 to 20 times higher than that of methanol, thus avoiding the formation of toxic metabolites. In the ethanol administration schedule, the goal is to maintain serum levels between 1 and 1.5 g/L. It should be administered as a bolus loading dose of 0.6 g/kg followed by a maintenance dose of 66–154 mg/kg/h. IV administration of ethanol is safer than oral administration, although it can produce irritation and thrombophlebitis [5, 6].

There is also the 4-methylpyrazole (4-MP) (fomepizole) antidote, which acts similarly to ethanol by competitively inhibiting ADH. The advantages with respect to ethanol are various, and its affinity for the enzyme is higher. It has a long half-life (meaning a long duration of action and dosing convenience), and it does not require continuous monitoring of plasma levels. It has fewer side effects. Its use in children has been described in [6, 7].

The purpose of this paper was to report the possibility of poisoning of children with accidental ingestion of methanol in perfumes and other household products that may contain this substance.

## Figures and Tables

**Table 1:** Evolution of acid base status.

	PO_2_ mmHg	PCO_2_ mmHg	HCO_3_ mEq/L	Sodium mEq/L	Potassium mEq/L	Chloride mEq/L	Anion gap
pH blood							
6.9	108	26	3	133	6	107	23
6.8	85	21	6.7	131	5.6	108	16.3
Hemodialysis							
7.1	85	22	6.4	131	5.9	108	16
7.4	95	45	23	140	4	103	14

**Table 2:** Evolution of laboratory tests.

Days	1	3	4	5
Urea mg/dL	33	18	—	—
Creatinine mg/dL	0.6	0.5	—	—
Albumin g/L	—	3.7	4.1	4.4
Glucose mg/dL		94		
AST UI/L**	4490	2942	1225	21
SGPT UI/L**	8030	924	248	37
Lactate dehydrogenase UI/L	2609	5600	2800	800
Blood pH	6.9	7.34	7.38	7.4
HCO_3_ mmol/L	3	24	23	24
Methanol mg/dL*	1.47	—	0	0

*Toxicological examination: spectrophotometer method.

**Aspartate aminotransferase.

**Glutamic pyruvic transaminase.
